# The Landscape of Tumor-Specific Antigens in Colorectal Cancer

**DOI:** 10.3390/vaccines8030371

**Published:** 2020-07-10

**Authors:** Nurul Ainaa Adilah Rus Bakarurraini, Nurul Syakima Ab Mutalib, Rahman Jamal, Nadiah Abu

**Affiliations:** UKM Medical Molecular Biology Institute (UMBI), Universiti Kebangsaan Malaysia, Kuala Lumpur 56000, Malaysia; ainaaadilah92@gmail.com (N.A.A.R.B.); syakima@ppukm.ukm.edu.my (N.S.A.M.)

**Keywords:** neoantigens, frameshift peptides, immunotherapy, vaccines, adoptive T cells

## Abstract

Over the last few decades, major efforts in cancer research and treatment have intensified. Apart from standard chemotherapy approaches, immunotherapy has gained substantial traction. Personalized immunotherapy has become an important tool for cancer therapy with the discovery of immune checkpoint inhibitors. Traditionally, tumor-associated antigens are used in immunotherapy-based treatments. Nevertheless, these antigens lack specificity and may have increased toxicity. With the advent of next-generation technologies, the identification of new tumor-specific antigens is becoming more important. In colorectal cancer, several tumor-specific antigens were identified and functionally validated. Multiple clinical trials from vaccine-based and adoptive cell therapy utilizing tumor-specific antigens have commenced. Herein, we will summarize the current landscape of tumor-specific antigens particularly in colorectal cancer.

## 1. Background

Colorectal cancer (CRC) is a collective term that represents both colon and rectal cancers. According to the statistical data from the International Agency for Research on Cancer (IARC) of the World Health Organization (WHO), CRC is the third-most-frequently-diagnosed cancer around the world, with 1.85 million new cases every year, followed by lung and breast cancers [1]. CRC is also the third-most-common cancer in men after lung and prostate cancers [1]. Based on the latest revised statistical data from the American Cancer Society in 2020, the five-year relative survival rate of CRC is approximately 63%, whereas 89–90% survival rate was observed in patients with regional cancer. However, a drastic drop in survival rates was seen in CRC patients with distant cancer types which occur in around 14–15% of the patients.

Unfortunately, CRC is mostly asymptomatic—especially in patients with early stage disease [2]. Symptoms abruptly appear in the advanced stages of III and IV which include abdominal discomfort, diarrhea or constipation, cramping, loss of weight despite a good appetite, colorectal bleeding, muscle weakness and fatigue [2]. One therapeutic approach for cancer therapy that is increasingly being studied and developed is personalized immunotherapy treatment. The general premise of cancer immunotherapy aims to utilize the body’s immune system to fight against cancer cells [3]. To date, three categories of immunotherapy have been established which are; immune checkpoint inhibitors (ICIs), adoptive T cell therapies (ACTs) and cancer vaccines [4,5].

Among the immune cells that are being studied for personalized immunotherapy, CD8+ T cells are the most preferred immune cell to effectively target cancer cells [6]. For CD8+ T cells to have an effector function, its specificity towards tumor antigen is induced beforehand, in a process called T cell priming, mediated by antigen-presenting cells (APCs) such as dendritic cells (DCs) [7]. This T cell–APC interaction uses receptor-ligand signaling that depends on the projection of target antigen bound on MHC class I of APC [6]. Upon primed, the activated CD8+ T cells or CTLs can perform cytotoxic activity against the specific antigen-bearing tumor cells [8]. The series of events that begin with cytotoxic activation of naive T cells by DCs and end with tumor cytolysis response by cytotoxic T lymphocytes (CTLs) is also known as the tumor immunity cycle [9]. Following CTLs activation, the cells infiltrate into tumor deposits where they exert antitumor activity [8]. Infiltrating CTLs uses two pathways to mediate tumor cytolysis, which is Fas ligand (FasL)—mediated apoptosis and granule exocytosis pathways [10]. The binding of FasL of CTL to the Fas protein on tumor cells initiates a caspase cascade that eventually leads to cellular apoptosis [11]. Activated caspase-8 induces tumor apoptosis using two different apoptosis mechanisms. The first mechanism involves the degradation of a distinct substrate by procaspase-3 leading to cellular apoptosis, while the second mechanism activates the mitochondrial apoptotic pathway [12]. On the other hand, granule exocytosis pathway involves the release of two granzymes, A and B by CTLs upon reaching the tumor location [13]. A protein called perforin (PFN) mediates the trafficking of the granzymes into the target cells by forming pores in plasma membranes, thus providing the paths for the granzyme to diffuse into tumor cells [10]. It has been demonstrated that one of the roles of PFN is to induce tumor apoptosis [14]. These released granzymes enter the tumor cells and induce multiple processes such as cytolysis, extracellular matrix degeneration, inflammation and extravasation [15]. The understanding of the role of cytotoxic T cells in tumor regression allows researchers to investigate and improve the efficacy of T cells in treating cancer.

## 2. Immunotherapy in CRC-A Brief Overview

All immunotherapy approaches use the basic interaction of cell surface molecules called antigens in their mechanism of action [16]. ICIs are the proteins or antibodies that release the immune system’s brakes from the killing of cancer cells [17]. The basis of ICI development is derived from the fact that tumor cells express certain ligands to stop T cells from killing. This negative feedback of immune response is mediated by the binding of the programmed death receptor, PD-1 expressed on T cells with the ligand, PDL-1 expressed on tumor cells, thus shutting down the cytotoxic and anti-metastatic ability of the T cells [18]. This setback was finally overcome with the breakthrough discoveries of inhibitory agents that block the interaction. Two notable award-winning discoveries were accomplished by Tasuku Honjo and James P. Allison with the introduction of anti-PD1/PDL-1 and anti-CTLA-4 therapies, respectively [19]. The interest to discover further inhibitory agents intensified as more discoveries of other inhibitory receptors were made. Eventually, the first ICI, an anti-CTLA4 blocking antibody named ipilimumab (Yervoy) was approved by the Food and Drug Administration in 2011 to be used in the regiment to treat metastatic melanoma [20]. Treatments of cancer using ICIs have shown to yield successful tumor regression and improved prognosis in treated individuals [21]. It was reported that the use of ICIs in combined immunotherapy of ipilimumab and a PD-1 blocking antibody named nivolumab achieved complete remission in a patient with advanced liver, lung and lymph nodes metastasis [22]. In CRC, common ICI drugs used include nivolumab and ipilimumab, where combined drug therapy also showed a better prognosis than individual treatments [23].

Another notable immunotherapy strategy is known as cancer vaccines. Unlike normal vaccination, cancer vaccine is introduced as either early cancer prevention or during cancer treatment. Human papillomavirus (HPV) and Hepatitis B virus (HBV) vaccines are two types of vaccines that aim to prevent cancer formation. The relevance of the HPV vaccine was supported by the recent reports received from the four years of follow-up data showed reduced HPV-related carcinoma after the introduction of the vaccine [24]. Meanwhile, therapeutic cancer vaccines are usually immune boosters that aim to increase the efficacy of the body’s immune response in combating cancers. To some extent, cancer vaccines are also targeted to regulate the interaction between APCs and T cells [25]. For example, a well-known therapeutic cancer vaccine named Sipuleucel-T is a form of dendritic cell (DC) vaccine that induces immune response targeting tumor specific antigen (TSA) in prostate cancers simultaneously regressing tumorigenesis [26]. In non-small cell lung cancer (NSCLC), Stimuvax is an investigational cancer vaccine that stimulates the immune response to target Mucin 1 (MUC1) [27]. In CRC, vaccines against selected peptides such as Carcinoembryonic antigen (CEA) and MUC1 have been developed and have entered clinical trials [25,28]. Vaccination aims to enhance peptide spreading, in which the activated immune response against a single tumor associated antigen (TAA) could initiate secondary response towards other endogenous epitopes. This aim if achieved, will result in the activation of an immune response against multiple epitopes through a single immunization [29].

In addition to that, the application of ACT using T cells is an increasingly recognized area as a promising approach for personalized cancer treatment. ACT involves the generation of antigen-specific T cells either isolated or engineered, cultured in ex vivo environment and the reintroduction of the T cells into patients’ bodies [30]. A study done by Besser and colleagues on metastatic melanoma patients revealed excellent clinical responses using Young-tumor infiltrating lymphocytes (Young-TILs), in which 50% of the responders achieved objective response including two complete remission cases [31]. Further work on metastatic melanoma revealed complete responses in patients treated with ACT [32]. In CRC, it has been reported that the introduction of combined chemoimmunotherapy using activated αβ T cells together with the capecitabine–oxaliplatin (XELOX)-bevacizumab regimen achieved an 80% response rate with 26.7% complete response and 0% disease progression in stage IV CRC patients [33]. This shows that the advancement in molecular techniques has allowed for the identification of immunogenic antigens with more classifications.

## 3. Tumor Antigens

### 3.1. Tumor-Associated Antigens vs Tumor-Specific Antigens

Antigens are proteins that are found on the cell surface of every cell. Antigens derived from normal DNA expressions in healthy cells are not immunogenic and therefore do not trigger immune responses. However, antigens derived from foreign DNA of viruses and bacteria, as well as mutated DNA, are able to trigger cytotoxic T cell responses against the antigens [34]. In cancer, gene expressions derived from the mutated DNA segments may give rise to mutated proteins which will be presented as an antigen on tumor cells. These antigens can trigger T cell recognition resulting in an anticancer response. In 1992, an antigen that was specifically recognized by an autologous CTLs was found in human melanoma cell lines [35]. Research progress on identifying cancer antigens escalated rapidly with subsequent discoveries of immunogenic antigens expressed in human melanoma and breast cancer cases [36,37]. In the context of immunotherapy, the attempt to identify immunogenic antigens is especially critical. To date, two major classifications of cancer-produced antigens, tumor-associated antigens (TAA) and tumor-specific antigens (TSA), have been determined based on their tissue type location, ability to trigger an antitumor immune response and production consistency in the tumor than normal cells [38].

TAAs are derived from the expression of normal genes in both normal and tumor cells. TAA can either be any glycoprotein or protein synthesized by the tumor cells [39]. Despite being presented on a normal cell’s surface, TAA’s immunogenicity arises from the differential expression level in tumor cells where its production is relatively higher. Therefore, its presentation on the tumor cell surface can inaugurate a CTL response. An example of TAAs is the cancer/testis antigen (CTA) which was discovered in 1997 from serologic analysis of tumor mRNA and the autologous patient’s serum [40]. This discovery had provided the first evidence of differential mRNA expression level of the antigen which showed remarkable elevated expression level in testicular and ovarian cancer cells than normal cell types [40]. In CRC for instance, ten CTA genes were detected with differential expression frequencies with NY-ESO 1 expression exhibited antibody-positive response [41]. On top of this, it was found that a significant level of expression of the CTA gene, MAGEA3, was present in most CRC samples [41,42]. A more recent study by Soh et al., revealed PASD1, a type of CTA, was able to elicit CTL response in CRC cells expressing the protein [43]. Although CTAs have shown some moderate success, concerns still arise due to the presence of these antigens on normal cells, which reduces its specificity [44].

Another notable cancer antigen is called TSA or neoantigen. Neoantigens are derived from the expression of non-synonymous somatic mutations including insertions/deletions (indels), frameshift mutations, single nucleotide variations (SNV), structural variations and fusion genes occurring in cancer cells [34]. Since tumorigenesis is generated from DNA mutations of the cells that lead to dysfunctional peptides synthesis, neoantigens are only present on the tumor cell surfaces and are absent on the normal cell surfaces [45]. This specificity becomes an ideal attribute for the development of adaptive immune therapy in treating cancers. Several reports have discovered autoimmune toxicity as well as central immune tolerance of cytotoxic T cells against TAAs due to their presentations on normal cell surfaces [45,46]. As a result, the development of cancer vaccines using TAAs as cytotoxic baits is not as promising than the use of neoantigens. Research on neoantigens have provided a potential data in which several numbers of studies have validated these neoantigens as selectively immunogenic.

### 3.2. Biosynthesis of Neoantigens

The biosynthesis of a neoantigen begins after the translation of abnormal peptides in tumor cells from the mutated DNA regions. These abnormal peptides are known as destructive ribosomal products (DRiPs) [47]. In the upstream process, it begins with DRiPs being subjected to cytosolic degradation mediated by small regulatory proteins known as ubiquitin. The binding of ubiquitins on DRiP structure acts as a recognition signal for proteasome to initiate the cleaving process [48]. This collaboration known as the system (UPS), performs to rapidly eliminate any abnormal peptides originating from defects in transcription and translation processes, DNA mutations, failures in folding and post-synthetic alterations [49,50]. Proteasomic cleaving in the cytosol leaves multiple short fragment products of oligopeptide around 2–20 amino acid residues in length [51]. Unfortunately, most of these peptide fragments are unable to survive the rough cytosolic environment full of peptidases and will be degraded as waste products. However, some will escape this fate and eventually successfully make contact with MHC class 1 molecule in the ER [52].

For the union to be possible, these chosen peptides must find their way into the ER for the assembly process with the MHC class 1 molecule. This goal is achieved with the help of a protein transporter associated with antigen processing (TAP). TAP is a transmembrane heterodimer protein consisting of two subunits called TAP1 and TAP2. This protein provides a channel for peptide fragments to enter the lumen of the ER, allowing the assembly of the peptides with MHC molecules [53]. In parallel with the TAP transport, the synthesis of MHC class 1 takes place in the lumen of the ER. The synthesis and structural folding are completed and facilitated by in-house chaperone proteins such as ERP57 and calreticulin that bind to the MHC class 1 molecule, forming a complex [54]. Together, the complexes travel towards the location of bottleneck traffic of peptide fragments. At this point, the attachment of peptides on MHC class 1′s binding pockets occur rapidly and efficiently. Finally, an MHC class 1 molecule with peptide presentation dissociates from TAP and other chaperone proteins and is transported out of ER to the Golgi apparatus, and subsequently to the cell membrane for antigenic presentation, which will then be recognized by cytotoxic T cells for rapid immune response activation [53].

## 4. Neoantigen Studies in CRC

### 4.1. SNVs-Derived Neoantigens

**Figure 1:**
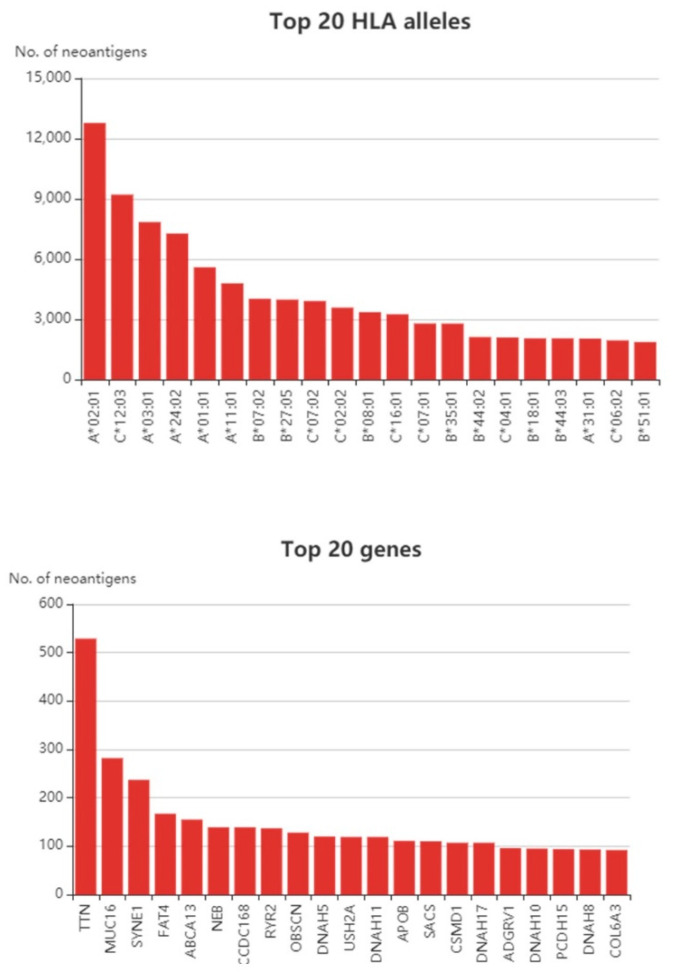
Top 20 human leukocyte antigen (HLA) alleles and top 20 neoantigen-producing genes in colorectal cancer (CRC) samples from the Cancer Genome Atlas (TCGA) cohort. Data obtained from the TSNAdb database [60].

At present, neoantigen-based studies in CRC have gained greater interest over the years. We examined The Cancer Genome Atlas (TCGA) cohort and utilized the Tumor-Specific Neoantigens DataBase (TSNAdb) to find out how many neoantigens can be predicted in CRC (Figure 1). It is not surprising that the TTN gene possessed the highest numbers of predicted neoantigens in CRC given the fact that it is a large gene [55]. Likewise, the TTN gene also yields among the highest number of neoantigens in breast cancer [55]. The TTN encodes a structural protein, and although its biologic role in relation to cancer is still debatable, some studies have shown that the TTN gene is immunologically relevant. For instance, it was shown that TTN mutations were able to predict better prognosis in response to immune checkpoint blockades in different solid tumors [56]. Concerning antigen-based studies, a study by Parkhurst et al. showed the mutated TTN peptide was able to induce CD8+ T cell response, but at a lower rate compared to the other tested antigens [57]. Apart from TTN, the MUC16 gene was also predicted to give rise to a high number of neoantigens. Besides CRC, other cancers were also predicted to produce a higher number of neoantigens towards the MUC16 gene such as gastric cancer [58] and pancreatic cancer [59]. It was discovered that MUC16 neoantigens were able to elicit CD8+ T cell response in long-term survivors of pancreatic cancers [59]. The study utilized two neopeptides from the MUC16 gene, MGKSTHTSM and VMKHLLSP peptides [59].

A study by Mennonna et al. identified new somatic mutations in CRC samples via high throughput sequencing [61]. Besides the commonly mutated genes found in CRC, the researchers discovered new mutations in the SMAD4 gene. The SMAD4^V370A^ epitope was found to be immunogenic in vivo—and on top of this—circulating T cells targeting this specific mutation was found present in the autologous T cells of patients harboring this mutation [61]. Apart from using patients’ tissue for neoantigen identification, patient-derived organoids have also been utilized [62]. Data on mass spectrometry analysis obtained by Newey and colleagues validated three neoantigens out of 612 non-silent mutations analyzed, which were peptides from the U2SURP, MED25 and FMO5 genes with peptides generated having 8–11 amino acids in length [62].

### 4.2. Frameshift/Indels-Derived Neoantigens in MSI CRC

The introduction of ICIs such as anti-CTLA-4, anti PD1-PDL1 as part of the cancer treatment regimen has resulted in a significant improvement in the outcomes of several cancer types [17]. It was highlighted that a significant level of cancer regression was detected in response to the treatment especially in melanoma and non-small cell lung cancer cases (NSCLC) [63,64]. The efficacy of this treatment alongside chemotherapy, however, is limited to some cancer types only. Patients with microsatellite stable (MSS) and proficient mismatch repair (pMMR) in CRC and ovarian cancer did not benefit much from this treatment [65,66]. However, it was reported that CRC patients with low mismatch repair (MMR) rate and high microsatellite instability (MSI) exhibited better prognosis when subjected to ICI treatment than the individuals with MSS and pMMR [66,67,68]. This finding highlighted several possible benefits of exploiting the information of the patient’s chromosome instability as a novel approach towards effective treatment. Tumor with high mutational burden is known to have a deficiency in mismatch DNA repair (dMMR) mechanism leading to high MSI. Moreover, it was reported on the higher efficacy of ICI treatments on MSI-high CRC than microsatellite stable (MSS) CRC [23].

CRC with MSI constitutes about 15% of all CRC cases inclusive of Lynch syndrome [69]. Since a better prognosis was observed in this type of CRC, frameshift mutations (FSMs) in the genome may cause the synthesis of tumor-specific neoantigens that are able to induce anti-tumor response [67]. The MSI status of CRC tissues eventually affects the number of mutations present and subsequently the number of possible neoantigens being processed. For instance, based on the TCGA dataset, as obtained from the Cancer Immune Atlas, CRC with MSI had significantly higher numbers of mutations and predicted neoantigens than the MSS group. This was also shown in a panel of CRC cell lines, where Rospo et al., showed that cell lines with MSI had higher loads or mutational burdens than MSS cell lines and cell lines carrying mutations in POLE genes [70]. In MSI tumors, the percentage of tumor-infiltrating lymphocytes (TIL) is higher when compared to MSS tumors, and this is due to the presence of increased frameshift mutations [71,72]. These frameshift mutations may yield immunogenic frameshift mutated-derived neoantigens that could attract higher CD8+ T cell density [73]. A pilot study by Tougeron et al. showed that MSI CRC tumors had the highest mutations in these four genes: ACVR2, TAF1B, ASTE1/HT001 and TGFBR2. Furthermore, they also showed that the highest density of TILs was associated with the presence of mutations in ASTE1/HT001 and PTEN [72]. This was in concordance with a study by Maby et al. that showed the TILs density increased when the mutations were present in the ASTE1, HNF1A or TCF7L2 genes [71]. This study also showed that MSI-CRCs have different immune microenvironments comprising of tumor neoantigen-specific CD8 T cells, which could be used to further advance immunotherapy in this specific subset of tumors, including in Lynch syndrome [73]. Lynch syndrome patients usually bear MSI-high type of CRC. Therefore, this subclass of patients may benefit more from neoantigen-based therapy. Furthermore, a study by Chang et al., showed Lynch syndrome polyps immune profile was indeed dynamic and had a higher CD4+ T cell count [74]. In terms of the neoantigen profile, the authors discovered that the Lynch syndrome polyps possessed low mutational burdens and subsequently low neoantigens, however, they discovered another subset of Lynch syndrome polyps that were hypermutated, yielding in higher neoantigens [74]. The hypermutated subset of Lynch syndrome polyps was similar to hypermutated tumors producing neoantigens involved in DNA damage response such as ataxia–telangiectasia mutated (ATM) and breast cancer type 1 (BRCA1) signaling pathways [74].

Studies on frameshift-derived peptides (FSP) in MSI-CRC have been performed over the past few decades. One of the earliest work was done on the TGFBRII gene in microsatellite instable (MSI) CRC. The TGFBRII is mutated in around 90% of CRC tissue with MSI, and often causing deletions or insertions [75]. The researchers generated several peptides corresponding to the mutated region of the gene and performed T cell-based assays [76]. The peptide p538 was found to have the highest immune potential. This was evidenced by the ability of tumor-infiltrating lymphocytes to react to p538, and also the generation of T memory cells from patients with MSI [75]. In the same study, the researchers also tested for Bax-derived mutated peptides, but no positive results were observed [75]. Following up from that, the same group was able to perform further in vitro assays and showed that upon repeated stimulation of T cells with antigen-presenting cells pulsed with the same TGFBRII peptide, the T cells were able to kill cell lines containing the mutant peptide [76]. Interestingly, a different group from Germany also performed FSP-based studies on several genes including TGFBRII in MSI-CRC [77]. This particular study focused on three different peptides deriving from TGFBRII mutants and they successfully showed that T cells induced FSP02 peptide were able to lyse cell lines harboring the mutant in an HLA-A201 dependent manner [77]. In addition to that, the immunogenicity of FSPs derived from TGFBRII was also proven in an in vivo model, and subsequently in clinical trials [78].

Apart from TGFBRII, other FSPs from mutated genes were also tested for such as OGT, HPDMPK, D070, U79260, FLT3 L among others [77]. The same study showed that FSPs derived from OGT and U79260 were also immunogenic. Nevertheless, although these FSPs were able to induce high levels of IFN-G as shown by the ELISpot assay, the T cells-pulsed with the same FSPs were unable to positively lyse respective mutation-carrying cell lines [77]. Interestingly, the same group repeated the study on the FSP-derived from OGT using different cell lines (positively carries the mutation and HLA-A201), CTLs pulsed with the same peptides were able to lyse the selected cell lines carrying OGT mutated proteins [79]. On top of this, this group also identified several other FSPs deriving from different genes such as caspase-5, TAF-1b and HT001 [80]. Out of the five FSPs tested, the authors identified an FSP from caspase 5, with the peptide sequence FLIIWQNTM, that was able to induce CTL-mediated lysis in an HLA-A201 manner [79]. Additionally, immunogenic FSPs from the MSH03 gene were also identified [81]. Garbe et al. tested 12 different FSPs and identified two HLA-A0201-restricted cytotoxic T cell epitopes from the MSH03 gene [81]. Furthermore, a novel HLA-A0201-restricted cytotoxic T cell epitope from the mutated region of U79260(FTO) was also discovered [82]. Other studies have also been conducted on MSI-CRC, including a study by Ishikawa et al. which identified an antibody that could react towards FSP derived from the CDX2 gene [83]. Speetjen et al. conducted a larger scale study using frameshift-mutated antigens common in MSI cancers including colorectal, gastric and endometrial cancers [84]. This study identified eight frameshift-mutated antigens that could be of interest, from different genes including TGFβR2-1, MARCKS-1, MARCKS-2, CDX2-2, TAF1B-1, PCNXL2-2, TCF7L2-2 and Baxα-1 [84]. Table 1 summarizes some of the mutated antigens that have been studied in CRC.

### 4.3. Fusion Genes-Derived Neoantigens

To date, most neoantigen-based research has focused on missense mutations to identify immunogenic epitopes. Fusion genes provide an attractive source of neoantigens, especially out-of-frame gene fusions [86]. This is because the presence of a novel out-of-frame sequence may result in a new open-reading frame and subsequently a long sequence of novel peptides that may potentially be immunogenic [86]. A recent study by Rasthe et al. has identified fusion genes-derived neoantigens from osteosarcoma [86]. This study optimized a bioinformatics pipeline to identify fusion transcripts with potential neoepitopes [86], In a different study by Yang et al., they identified DEK–AFF2 fusion-derived peptide that elicited T cell response in head and neck cancers with low tumor mutational burden [87]. With regards to CRC, it has been shown that MSS-CRC carries higher fusion genes than MSI-CRC, the same pattern of expression was also seen in stomach cancer and endometrial cancer [88]. Moreover, it has been shown that candidate neoantigens from fusion genes were more immunogenic than SNV and indel-derived candidates [88]. Notably, in the TCGA cohort, candidate neoantigens with the highest immunogenic potential were derived from gene fusions, thus making them better candidates for cancer vaccines [88].

### 4.4. Shared Neoantigens in CRC

Besides looking at patient-specific derived neoantigens, another approach is by targeting recurrently mutated genes that may yield neoantigens. Several studies have shown that common mutations by SNVs are also able to give rise to detectable neopeptides and elicit a specific immune response. For instance, a recent study by Iiizumi et al. revealed that some of the commonly mutated genes such as KRAS^G12^ and KRAS^G13^ were able to stimulate the immune response in healthy PBMCs [89]. They showed that the generated peptides were able to induce human leukocyte antigen (HLA) class II-restricted CD4+ T cell responses [89]. Similarly, Veatch et al. discovered that CD4+T cells in some non-small cell lung cancer patients were able to recognize the KRAS^G12 V^ mutated peptide instead of the wild-type peptide [90]. This was also in agreement with another study conducted by Quandt et al., where they generated long peptides comprising of common mutations in KRAS and TP53 and found higher effector T cells targeting gastrointestinal tumors bearing these mutations [91]. Interestingly, a study by Lo et al., discovered that tumor-infiltrating lymphocytes of a metastatic CRC patient were able to recognize the TP53^R175H^ mutated peptide—and when the T-cell receptor region was identified—the TCRs were able to recognize other types of cancers bearing the same mutation [92]. These studies showed that common mutations may be more immunogenic than initially regarded and this opens a new avenue of a universally targeted therapy to a wider cohort. Table 2 summarizes some of the hotspot mutations in CRC that may result in neoantigens and some of them were proven to be indeed immunogenic.

## 5. Clinical Trials Utilizing Tumor-Specific Antigens in CRC

Based on the database at clinicaltrials.gov, we found several completed and ongoing clinical trials that utilize mutated antigens/peptides for the treatment of CRC. The clinical trials ranged from vaccine-based therapies to T cell-based therapies. Among the completed studies is the clinical study NCT01461148, where the researchers tested vaccination in the form of FSPs derived from the *AIM2, HT001, TAF1B* genes for MSI-H CRC patients. This study was completed in 2015, and although there is a publication linked to this clinical trial, no results were obtained pertaining to the outcome of the therapy. Another clinical trial, NCT02600949, utilized a personalized peptide vaccine to treat pancreatic cancer and CRC. The treatment will be given together with pembrolizumab and imiquimod. As of this point of writing, this trial is still active, but is not recruiting anymore. A recent clinical trial, NCT04117087, described the use of the mutant KRAS-targeted long peptide vaccine in combination with monoclonal antibodies, Nivolumab and Ipilimumab for pMMR CRC patients. This study has not started recruitment and is expected to complete in 2024.

For clinical trials using T cell-based therapy, a clinical trial with the identifier NCT03431311 was executed to use TCR-engineered T cells against TGFBRII frameshift peptide in MSI CRC. However, the clinical trial was terminated one year after the first posting. Another clinical trial using adoptive cell therapy, NCT02757391, also utilized personalized immunotherapy. According to the trial, CD8+ T cells will be isolated from the patients and subjected to the immune response by selected personalized peptide antigens. This study combined CD8+ T cell therapy with the monoclonal antibody, Pembrolizumab. At the time of this writing, the clinical trial is active, but has stopped recruitment, with results expected to be available in 2025. Another recent clinical trial using T cell therapy is NCT03970382. This still is in the recruitment phase and will utilize neoantigen-targeting T cells in combination with nivolumab. Overall, Table 3 summarizes clinical trials that utilized mutated antigens in their studies.

Since most of the listed clinical trials have not generated any results yet, we took a look at other clinical trials utilizing neoantigens that have completed. In a clinical trial for glioblastoma, the phase I trial GAPVAC-101 of the Glioma Actively Personalized Vaccine Consortium (GAPVAC), the trial utilizes neopeptide vaccine in 15 glioblastoma patients [93]. This trial utilized a transcriptomic and peptidomic approach to generate personalized antigens. The outcome of this study showed that unmutated vaccines were able to induce CD8+ T cells, while the neoepitope-based vaccine, induced CD4+ T cell’s response against predicted neoepitopes [93]. In a separate clinical trial by Keskin et al., glioblastoma patients were also given neoantigen-based vaccines [94]. This study showed that the introduction of personalized vaccines was able to trigger the production of neoantigen-specific T cells in the tested patients [94]. This trial showed that the administration of neoantigen-based vaccine was also effective in tumors with a low mutation burden [94]. Interestingly, the reaction of CD4+ T cells was more prominent in this clinical trial even though the prediction against HLA type 1 was used [94].

## 6. An Overview of Common Platforms and Protocol for Neoantigen Prediction

It is well-established that the application of T cell therapy targeting neoantigens indeed imparts clinical benefits in cancer patients. Earlier studies on neoantigen identification showed that the combination of bioinformatics analysis, genomics and immunological approaches massively improved the fidelity of the whole process. Robbins and Rosenberg utilized whole-exome sequencing data coupled with the MHC class 1 binding algorithm and successfully identified mutated protein expressed on melanoma cells that were recognized by adoptively transferred T cells [95]. Studies conducted by Fritsch et. al., identified several mutated peptides based on cDNA expression cloning or MHC–peptide elution and successfully validated several MHC binding prediction tools [96]. The experience and the confidence from multiple studies done over the years had led to the development of a standard workflow for rapid prediction and identification of putative neoantigens in human samples. The genome status of tumor CRC samples obtained from patients needs to be analyzed for its mutational status and MSI level. In this process, the next-generation sequencing (NGS) approach can be used to do rapid whole-genome sequencing (WGS) or whole-exome sequencing (WES) tests on tumor samples. Using massively parallel sequencing (MPS) approaches, both DNA and RNA-converted-cDNA can be used to generate a suitable library for sequencing [97]. WES uses designed probes to bind to the coding exons of the analyzed samples. The bound probes are then captured, isolated and denatured for further quantification and sequence analysis. Mutational detection is determined by aligning the sequence reads to reference genome i.e., genome of the paired-normal sample. Once the reads are aligned, multiple algorithms are used to interpret the variants obtained into types of mutations such as single nucleotide variants (SNV), deletion or insertion. Next annotation steps involve the conversion of the mutated DNA sequence into the amino acid sequence, thus providing the primary data to identify and sort neoantigens [97]. The validation of the expression of these mutated regions can be conducted using RNA-sequencing or mass spectrometry analysis. Referring to the work done by Fritsch, Robbin and Rosernbeg teams, current critical algorithms to predict epitopes are the ones with MHC class 1 binding prediction. Since the presentation of neoantigens is dependent on the expression of MHC class I molecules, it is especially important to analyze the peptide candidates based on the binding affinity towards MHC molecules. As such, several algorithms are used to establish this information including tools to perform HLA typing to determine the types of HLA/MHC of the samples, proteasomal cleavage prediction that predicts the end product sequences of the modified peptide candidates, TAP transport status to predict the efficiency of TAP transportation of MHC class I, and alongside with the binding prediction of peptide candidates on MHC binding pockets. The common tools used for every interpretation are summarized in Table 4 [98].

## 7. Conclusions and Future Perspectives

Apart from the above mentioned mechanism of neoantigen generation, other mechanisms have also shown to be able to produce neoantigens. For example, new candidates of transcripts may also be generated via alternative splice variants [105] or endogenous retroviral elements [106]. Although there are various mechanisms in the induction of neoantigens, the immunogenic potential may also differ. For instance, it has been shown that neoantigens deriving from indels are more enriched for mutant specific binding than SNVs [107], thus making it more likely to be immunogenic. Apart from that, SNV-based neoantigens have the advantages of being more well-studied, easier identification and validation, and a more well-established platform than other neoantigens [108]. Even though SNV neoantigens are highly patient-specific, other types of neoantigens such as alternative splice variants and fusion-gene antigens are shared among the subpopulation. This attribute makes them even more promising candidates as they can be used to cater for a wider target [108].

Most of the reported studies evaluated in this review were able to mount CD8+ T cell response against tumor-specific antigens. Nevertheless, other immune cells may also play important roles in inducing neoantigen-specific immune response such as CD4+ T cells and B cells [109]. In a recent study by Veatch et al., the researchers identified the presence of CD4+ T cells that were reactive to neoantigens in several NSCLC patients [90]. A similar observation was also seen in treatment naïve ovarian cancer patients by Liu et al. where the authors found subsets of CD4+ and CD8+ T cells that were reactive towards neoepitopes [110]. Interestingly, in some cases, although most cancer vaccines were designed against the MSI class 1 subtype, the response was more favorable towards CD4+ T cells [111]. In CRC, it has been reported that several mutated peptides were also able to elicit CD4+ T cell-specific response, apart from CD8+ T cells [61].

**Figure 2:**
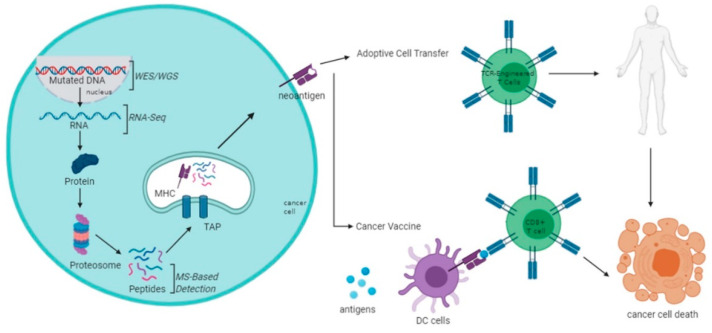
Brief schematic overview of neoantigen production and prediction and application through adoptive T cell therapies (ACT) and vaccination.

The future of personalized cancer treatment using tumor-specific antigens remains a challenge. However, the rapid expansion of knowledge in molecular and cell biology has led to more gaps being filled. The utilization of next-generation technologies and advanced bioinformatics have also contributed to the progress of personalized immunotherapy (Figure 2). Nevertheless, more challenges lay ahead and this needs to be addressed from time-to-time. Tumor heterogeneity is one of the biggest setbacks in implementing tumor-specific antigens-based therapy. Apart from CRC, most solid tumors are also heterogeneous, and the challenge posed by this characteristic needs to be overcome eventually. On top of this, personalized immunotherapy dedicating to one patient at a time is labor and cost-intensive. The search for the “one-size-fits-all” approach to immunotherapy is still one of the best ways to move forward, nevertheless, certain specificity is likely required for targeting different tumors. Validation of neoantigens is also imperative in identifying which of the identified antigens are truly functionally immunogenic. Furthermore, the applications of vaccine-based therapy and adoptive cell therapy have also been progressing well. Although one method may have some advantages or disadvantages over the other, the utilization of tumor-specific antigens may significantly contribute to personalized immunotherapy. Collectively, more research is needed to truly make use of tumor-specific antigens, especially in the CRC setting.

## Figures and Tables

**Table 1:** List of mutated antigens that were studied in CRC.

Source	Gene	Epitope	HLA	CD4+ or CD8+ T Cells	References
MSI-CRC	OGT	SLYKFSPFPL	HLA-A0201	CD8+	[79]
	CDX2	–	–	N/A	[83]
	U79260(FTO)	TLSPGWSAV	HLA-A0201	CD8+	[82]
	TGFβIIR	RLSSCVPVA	HLA-A0201	CD8+	[75,76,77,78]
	CASP5	FLIIWQNTM	HLA-A0201	CD8+	[80]
	CASP8	ELLVRINRL	HLA-B*08:01	CD8+	[85]
	MSH03	–	HLA-A0201	CD8+	[81]
	MARCKS-1, MARCKS-2 CDX2-2, TAF1B-1, PCNXL2-2, TCF7L2-2 Baxα+1	–	–	CD8+	[84]
CRC	SMAD4	–	–	CD8+ & CD4+	[61]
CRC Organoids	U2SURP	IQEERDERHKR	–	N/A	[62]
CRC Organoids	MED25	SVDANTTL	–	N/A	
CRC Organoids	FMO5	RYVENQRHTI	–	N/A	

**Table 2:** Recurrently mutated genes in CRC that may yield potential neoantigens.

Gene	The Overall, Prevalence in CRC	Mutation Hotspots	Mutation Yielding Possible Neoantigens	Reference/Database
KRAS	42%	G12, G13, Q61	G12A, G12C, G12D, G12R, G12S, G12 V, G13C, G13D, Q61H, Q61R, Q61 K	[60,89,90] TSNAdb, TCIA
BRAF	12%	V600	V600E	TSNAdb
TP53	60%	R175, R248, R273, R213, R282	R213 L, R213Q, R248Q, R248 W,	TSNAdb [91,92]
PIK3CA	28%	E545	E545 K	TSNAdb
FBXW7	17%	R465	–	TSNAdb

**Table 3:** List of clinical trials using tumor-specific antigens for CRC in their strategy.

Type of Therapy	Strategy	Combined Therapy	Number of Patients	Status	Trial Number
Vaccine	Personalized neoepitope yeast-based vaccine	–	16	Recruiting	NCT03552718
Peptide Vaccine	AIM2(-1)/HT001(-1)/TAF1B(-1) FSP vaccine	Montanide^®^ ISA-51 VG	22	Completed	NCT01461148
Peptide vaccine	Personalized Synthetic Tumor-Associated Peptide Vaccine	Imiquimod, Pembrolizumab	60	Active	NCT02600949
Peptide Vaccine	mutant KRAS-targeted long peptide vaccine	Nivolumab and Ipilimumab	30	Active	NCT04117087
Adoptive Cell Therapy	TCR-engineered T cells against TGFBRII frameshift peptide	–	1	Terminated	NCT03431311
Adoptive Cell Therapy	CD8+T cells against personalized peptide antigens	Pembrolizumab	1	Active	NCT02757391
Adoptive Cell Therapy	neoantigen-targeting T cells	Nivolumab	148	Active	NCT03970382

**Table 4:** Common tools used in neoantigen predictions.

No.	Tools	Interpretations
1.	OptiType [99]	HLA typing
2.	NetChop [100]	Proteasomal peptide processing
3.	NetCTL [101]	TAP transport, proteasomal processing, HLA binding
4.	NetMHC [102]	HLA binding
5.	NetMHCpan [103]	HLA binding across more HLA alleles
6.	pVAC-Seq [104]	RNA expression, HLA binding, DNA mutation
